# Development and *In-vitro* Evaluation of a Contraceptive Vagino-Adhesive Propranolol Hydrochloride Gel

**Published:** 2012

**Authors:** Elahe Tasdighi, Zahra Jafari Azar, Seyed Alireza Mortazavi

**Affiliations:** a *Pharmaceutical Sciences Branch, Islamic Azad University, Tehran, Iran.*; b *School of Pharmacy, Shahid Beheshti University of Medical Sciences, Tehran, Iran.*

**Keywords:** Propranolol HCl; Contraceptive gel; Mucoadhesive strength; Vagino-adhesive; In-vitro drug release; Sodium alginate.

## Abstract

The objective of the present investigation was to develop and evaluate a contraceptive vagino-adhesive propranolol hydrochloride gel. To achieve this, various mucoadhesive polymers including guar gum (1-4% w/w), sodium alginate (4-7% w/w), xanthan gum (2-5% w/w ), HPMC 4000 (3-5% w/w), Na CMC (4-7% w/w), carbomer 934 and carbomer 940 both in the range of 0.5-2.0% w/w, were dispersed in an aqueous-based solution containing the drug (1.6% w/w). The mucoadhesive properties of the gels were assessed on sheep vaginal mucosa (as model mucosa) in pH 4.5 citrate-phosphate buffer at 37°C. Formulations containing charged functional groups in their polymeric structure, showed higher mucoadhesive strengths in comparison to those composed of neutral polymers. *In-vitro* drug release profiles of the gels were determined in pH 4.5 citrate-phosphate buffer. Results indicated that, only formulation F_13_ (containing sodium alginate 6.5% w/w), could release its drug over 12 h, with a burst release at the initial phase followed by a sustained release pattern. This formulation, which showed a good mucoadhesive strength (386.97 ± 9.31 mN), was considered as the final formulation and underwent complementary tests including determination of drug content and duration of mucoadhesion. Its drug content was found to be 101.05 ± 0.106% (n = 3) and it attached to the model mucosa for more than 10 h. In conclusion, formulation F_13_ was considered as the most desirable formulation as it exhibited appropriate mucoadhesive properties while having the potential of providing an immediate contraceptive effect, followed by a prolonged drug release which is assumed to render longer contraceptive efficacy.

## Introduction

At the present time, one of the social problems regarding world health is the stability of population growth. Hence, the necessity to develop a safe, effective and affordable precoital spermicide contraceptive to control pregnancy and population growth still exists (1). Although many types of spermicidal contraceptives are available, they have side effects and are not easily accepted. The most widely available spermicide is the detergent nonoxynol-9 (N-9). This potent vaginal spermicide which is an organic surfactant, acts by disrupting the lipids within the sperm plasma membrane and causes rapid loss of sperm motility. However, nonoxynol-9 causes inflammation and genital ulcerations and thus increases the chance of HIV-1 infection, especially if used frequently. Therefore, vaginal spermicides lacking detergent-type membrane toxicity may offer a significant clinical advantage over the currently available detergent-type spermicides (2). 

Propranolol HCl is a *β*-adrenergic blocker that is used to treat tremors, angina, hypertension, heart rhythm disorders, *etc* (3). This drug, also possesses local anesthetic (or membrane stabilizing) activity of short latency and fairly long duration and is a potent inhibitor of human sperm motility *in-vitro* and has a similar effect *in-vivo* in rats (4). The concentration of propranolol hydrochloride, which inhibits sperm motility by 50% (IC50), is 0.3 mM. The local anesthetic properties of this drug is in fact, the underlying mechanism which is responsible for inhibition of sperm motility, rather than its *β*-blocking potential. These characteristics give a potential to this drug for use as a vaginal contraceptive or for incorporation in an intrauterine contraceptive drug delivery system, while being devoid of numerous adverse effects reported from use of other types of contraceptives (2).

The efficacy and tolerability of 80 mg propranolol hydrochloride tablets as vaginal contraceptives were studied in 198 fertile women for 11 months. The calculated one year life table pregnancy was 3.4 out of every 100 women. No major adverse effects were encountered (4).

Another study was conducted to observe the possible side effects of propranolol HCl tablets after insertion into the vagina. Six healthy women participated in this study. None of these women complained of vaginal irritation or other related symptoms and vaginal sensation was unaffected. No postural symptoms, breathlessness or wheezing were noticed (5).

The conventional formulations of vaginal drug delivery systems (VDDS) are associated with poor retention due to self-cleansing action of vaginal tract, leading to poor compliance. To overcome this problem, mucoadhesive polymers can be used for the preparation of VDDS (6).

Bioadhesion can be defined as the process by which a natural or a synthtetic polymer can adhere to a biological substrate. When the biological substrate is a mucosal layer, then the phenomenon is known as mucoadhesion (7). The vaginal route appears to be highly appropriate for mucoadhesive drug delivery systems, to retain systems for treating largely local (although some systemic) conditions, or for use in contraception (8). 

The main advantage of mucoadhesive systems is the possibility of increasing the time of residence *in-situ*, thus reducing the number of applications (9).

The most widely used mucoadhesive vaginal drug delivery systems are gels. Gels are semisolid, three-dimentional, polymeric matrices comprising small amounts of solids, dispersed in relatively large amounts of liquid, yet possessing more solid-like character. These systems have been used and are receiving a great deal of interest as vaginal drug delivery systems (8). Among vaginal formulations, gels are easy to manufacture, comfortable and have the ability to spread onto the surface of mucus and to achieve an intimate contact with vaginal mucosa. Moreover, because of their high water content and rheological properties, they present the further advantage of a hydrating and lubricating action, which is particularly useful in pathological situations characterized by dryness of vaginal mucosa. The degree of lubrication provided by a product, will likely be an important determinant of its acceptability and use (10, 11).

Although there are only a few acceptability studies in literature about vaginal drug delivery systems, most of them demonstrate that gels are among women’s preferences, concerning to vaginal formulation of choice (12).

One of the commercially available products combining spermicidal and microbicidal effects is Acidform^®^, an acidic vaginal gel originally developed by Program for Topical Prevention of Conception and Disease (TOPCAD). It adheres well to sheep vagina and cellophane *in-vitro*. In women, Acidform^®^ forms a thin bioadhesive layer of gel over the vagina and cervix, as observed colposcopically 12 h after use. Unlike N-9, Acidform^®^ gel was safe when applied vaginally during six consecutive days through colposcopic observation (13).

In a study by Saxena *et al*.(14), a biodegradable hydrogel composed of biocompatible matrices, impregnated with ferrous gluconate, ascorbic acid and mixtures of polyamino and polycarboxylic acid as pH modifiers; was prepared. Ferrous gluconate immobilizes sperm tail, ascorbic acid increases the cervical mucus viscosity and the pH modifiers, sustain the pH of the vagina close to 4.5. This combination ensures that sperms are immobilized and do not survive.

The success of propranolol hydrochloride as a novel, effective vaginal spermicide whose failure rate compares favorably with that of other methods of contraception, has already been described in the literature. This spermicidal action was observed with vaginal insertion of conventional tablets containing 80 mg propranolol HCl (4). These conventional tablets are associated with poor retention in the vaginal canal as well as lacking lubricating action observed with the vaginal gel application. Thus, in this investigation attempts were made to develop a vagino-adhesive propranolol HCl gel (containing 80 mg of the drug in each applicator) that not only provides lubricating effects but also by sustaining the drug release as well as adhering to the vaginal mucosa for an extended period of time, would ensure longer contraceptive efficacy. 

## Experimental

 Propranolol hydrochloride was purchased from Rouz Darou Pharmaceutical Co., Iran. Xanthan gum, guar gum and sodium alginate were obtained from Silverline Chemicals, India. Hydroxypropylmethyl cellulose 4000 (HPMC 4000) and sodium carboxymethyl cellulose (Na CMC) were from Shin-Estu Chemical Co., France. Carbomers including carbomer 934 (C934) and carbomer 940 (C940) were from BF Goodrich, Germany. Methanol, anhydrous citric acid, disodium hydrogen phosphate, propylene glycol and lactic acid were all purchased from Merck, Germany. 

*Preparation of propranolol hydrochloride gel formulations*

Different classes of excipients usually incorporated in order to prepare vaginal gels include gelling agents, humectants, preservatives and vehicles (8). For this purpose, different concentrations of various mucoadhesive polymers including the natural polymers guar gum (in the range of 1-4% w/w), sodium alginate (in the range of 4-7% w/w) and xanthan gum (in the range of 2-5% w/w), and semi-synthetic polymers HPMC 4000 (in the range of 3-5% w/w) and Na CMC ( in the range of 4-7% w/w), as well as the synthetic polymers C934 and C940 both in the range of 0.5-2.0% w/w; were utilized to develop the gels. In order to formulate the mucoadhesive gels containing the drug, gelling agent was dispersed slowly in an aqueous-based solution containing propranolol hydrochloride (1.6% w/w, as the active ingredient), propylene glycol (5.0% w/w, as humectant) and sodium benzoate (0.25% w/w, as antimicrobial preservative), with the help of an overhead stirrer. The pH of the vagina is maintained by lactobacilli which produce sufficient lactic acid to acidify vaginal secretions to pH 3.5-4.5. The pH is important in terms of design and the efficacy of drug delivery systems (11, 15). Hence, the pH of each formulation was adjusted to 4.0 (so as to be within the normal vaginal pH range) by the addition of lactic acid. Excipients are usually chosen from those materials which are deprived of therapeutic activity. Nonetheless, it is not always true; as it can sometimes be advantageous in the development of a pharmaceutical system (7). In this study, the main purpose of incorporating lactic acid into the formulations was its spermicidal activity (16). The composition of polymers within each of the gel formulations is given in Table 1. The prepared gel formulations were then tested on the basis of physical appearance, apparent viscosity, spreadability and strength of mucoadhesion. Then, four of these formulations were selected (named as chosen formulations) and underwent further examinations including determination of *in-vitro* drug release properties and drug release kinetic studies. Among these formulations, one formulation was selected as the final propranolol HCl gel formulation, which was then assessed in terms of complementary tests including propranolol HCl content within the gel as well as the duration of mucoadhesion.

**Table 1:** Polymer composition (% w/w) within the aqueous-based propranolol HCl gel formulations

**Formulation** **Code**	**Guar gum**	**Xanthan** **gum**	**Na alginate**	**Formulation** **code**	**HPMC 4000**	**Na CMC**	**C934 **	**C940**
F_1_	1.0	−	−	F_15_	3.0		−	−
F_2_	2.0	−	−	F_16_	4.0		−	−
F_3_	3.0	−	−	F_17_	4.5		−	−
F_4_	4.0	−	−	F_18_	5.0		−	−
F_5_	−	2.0	−	F_19_	−	4.0	−	−
F_6_	−	3.0	−	F_20_	−	5.0	−	−
F_7_	−	3.5	−	F_21_	−	6.0	−	−
F_8_	−	4.0	−	F_22_	−	7.0	−	−
F_9_	−	5.0	−	F_23_	−	−	0.5	−
F_10_	−	−	4.0	F_24_	−	−	1.0	−
F_11_	−	−	5.0	F_25_	−	−	2.0	−
F_12_	−	−	6.0	F_26_	−	−	−	0.5
F_13_	−	−	6.5	F_27_	−	−	−	1.0
F_14_	−	−	7.0	F_28_	−	−	−	2.0

*M* *easurement of spreadability of gel formulations*

The area of spreadability of each propranolol HCl gel formuation, was determined using the following technique: five hundred milligrams (0.5 g) of the gel formulation was placed within a circle of 1 cm diameter, premarked on a glass plate, over which a second glass plate was placed. A weight of 500 g was allowed to rest on the upper glass plate for 5 min. The increase in the diameter due to spreading of the gel was noted (17) and then the spreading area was calculated using Equation 1, representing the area of a circle. This test was performed in triplicate and the data obtained expressed as mean ± standard deviation (SD).

*A *= *π r*^2^ (Equation 1)

In the above equation, *A* is the area of the circle formed due to spreading of the gel (cm^2^), and *r* is the radius of the circle (cm).

*Assessment of the mucoadhesive strengths of the gels*

**Figure 1:**
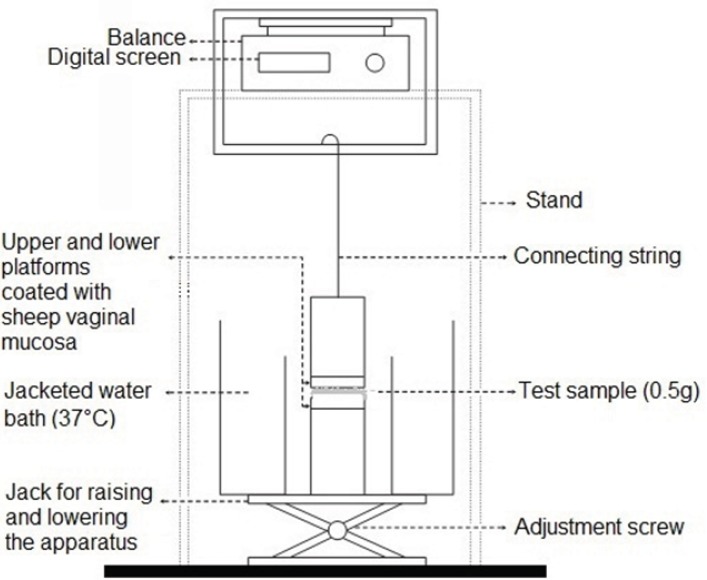
Schematic drawing of the apparatus used for assessing the in vitro mucoadhesive strength of propranolol HCI gel formulations

In order to evaluate the mucoadhesive strength of the prepared propranolol HCl gel formulations, the apparatus shown in Figure 1 was used. This apparatus was principally similar to those described in previous studies (18, 19). The upper stationary platform was linked to a balance, measuring the force needed to break contact between the gel and the mucosal membrane. The test cell was filled with pH 4.5 citrate-phosphate buffer, maintained at 37°C. Freshly removed sheep vaginal mucosa was used as the model mucosal membrane, and fixed in place over the two cylindrical platforms of the test apparatus and allowed to equilibrate in this solution for 2 min. Five hundred milligrams (0.5 g) of each gel formulation was then individually sandwiched between the two mucosa-covered platforms. Gels were kept in place for 5 min and then a constantly increasing force of 0.1 g/s was applied on the adhesive joint formed between the vaginal mucosa and the test gel, by gradually lowering the lower platform. This trend was continued until the contact between the test gel and the mucosa was broken and the maximum detachment force measured, was recorded. This force was taken as the strength of mucoadhesion of the test sample. Each experiment was run in triplicate, and results were expressed as mean ± SD. 

*Determination of in-vitro drug release profiles from the chosen propranolol HCl gel formulations*

The *in-vitro* release of propranolol HCl was determined from the chosen vaginal gel formulations using a dialysis tubing (MWCO of 12400 D; 99.99% retention, Sigma-Aldrich, USA) placed in the release medium under constant magnetic stirring. Five grams (5.0 g) of the gel formulations, were individually packed into sections of dialysis tubing (the length and the width of each section were 50 and 40 mm, respectively) with the ends being tightly fastened. The release medium was 200 mL of 0.1 M citrate-phosphate buffer (pH = 4.5). The medium was maintained at 37°C and stirred continuously at 100 rpm. Five mL (5.0 mL) aliquots of the release medium were withdrawn at predetermined time intervals and replaced by fresh citrate-phosphate buffer, to provide sink condition. Each withdrawn sample was further diluted with pH 4.5 citrate-phosphate buffer and it’s absorbance measured using uv-visible spectrophotometer (Shimadzu uv-visible 120A, Japan) at a λ _max_ of 289.2 nm. The absorbance was converted to drug concentration using the linear calibration curve constructed (Absorbance = 0.0196 Concentration (mg/L) – 0.0114; R^2 ^= 0.9995) and then cumulative percentage of propranolol HCl released was calculated with the help of a dilution factor. All measurements were performed in triplicate (n = 3).

*In-vitro drug release kinetic studies of the chosen propranolol HCl gel formulations*

In order to study the release kinetics of the chosen propranolol HCl gel formulations, data obtained from *in-vitro* drug release studies were fitted into different kinetic mathematical models. These models were as follows: zero order (Equation 2), as cumulative percentage of drug released *vs.* time, first order (Equation 3), as Log cumulative percentage of drug remaining *vs.* time, and Higuchi’s model (Equation 4), as cumulative percentage of drug released *vs.* square root of time. 

*Q = Q*_0_ *+ K*_0_ *t *(Equation 2)

Where *Q* is the amount of drug released,* Q*_0_ is the initial amount of the drug in the solution (it is usually zero), *K*_0_ is the zero order rate constant expressed in units of concentration/time and *t *is the time.

*LogC = LogC*_0 _–* K*_1_*t /2.303 *(Equation 3)

Where *C*_0_ is the initial concentration of the drug, *K*_1_ is the first order release rate constant and *t* is the time.

*Q*_t_ *= K*_H_ *t* ^1/2 ^ (Equation 4)

Where* Q*_t_ is the amount of drug released in time* t *and *K*_H_ is the Higuchi’s model release rate constant reflecting the design variables of the system (20). 

In order to evaluate the mechanism of drug release from the prpranolol HCl gel formulations, the first 60% drug release data were fitted in the Korsmeyer-Peppas model (Equation 5), as Log cumulative percentage of drug released *vs.* Log time.

*M*_t_ */M*_∞_ *=Kt* ^n^ (Equation 5)

Where *M*_t_* /M*_∞_ is the fraction of drug released at time *t*, *K* is the rate constant and *n* is the release exponent (20, 21). The *n* value is used to characterize different release mechanisms, as given in Table 2 for cylindrical shaped matrices.

**Table 2:** Release exponent and solute release mechanism for cylindrical shaped matrices

**Release exponent (n)**	**Overall solute release mechanism**
0.45	Fickian diffusion
0.45 <n < 0.89	Anomalous (non-Fickian) diffusion
0.89	Case-ll transport
n > 0.89	Super case-ll transport

*Determination of drug content within the final gel formulation*

For determination of drug content within the final propranolol HCl gel formulation) of the gel was weighed in a 100 mL volumetric flask and then, 10.0 mL methanol was added to it (17). The content of the flask was stirred vigorously until the gel got completely dispersed to give a clear solution. The volume was adjusted to 100 mL with citrate-phosphate buffer pH=4.5. The obtained solution was diluted appropriately (dilution factor = 10) by the addition of pH 4.5 citrate-phosphate buffer and absorbance was measured in a uv-visible spectrophotometer (Shimadzu uv-visible 120A, Japan) at λ _max_ = 289.2 nm.

The absorbance was converted to drug concentration, using the linear calibration curve mentioned earlier. Then, the exact amount of the drug in the tested gel formulation was calculated with the help of dilution factor. This test was performed 3 times and the mean value ± SD was calculated.

**Figure 2:**
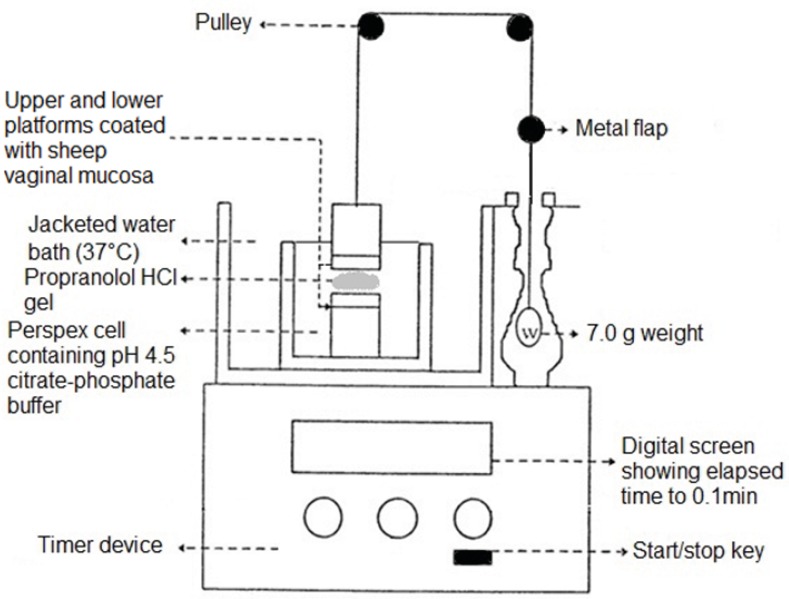
Schematic drawing of one compartment of the apparatus used for assessing the duration of mucoadhes on of the final propranolol HCI gelformulation (F_13_).

*Determination of duration of mucoadhesion of the final formulation*

The apparatus used for this study was based on that described in previous studies (19, 22). The test apparatus (Figure 2) was composed of six upper and six lower cylindrical platforms within a clear jacketed perspex cell, filled with pH 4.5 citrate phosphate buffer. Freshly removed sheep vaginal mucosa (used as the model mucosal membrane) was mounted securely in place, mucosal side up-wards, on each of the platforms and allowed to equilibrate for 2 min. The test gel was then sandwiched between the two platforms and allowed to stand for 5 min. Next, through two pulley systems, a 7.0 g weight was applied on each upper platform (this weight was chosen through initial studies). As soon as the contact between the test gel and the mucosal surface broke, a small flap dropped onto a photocell detector, stopping the timer device (recording the elapsed time to 0.1 min) and measured the duration of mucoadhesion of the gel.

*Statistical analysis*

Data obtained from spreadability and strength of mucoadhesion of propranolol HCl gel formulations, were analyzed using the one way ANOVA and Tukey post-hoc test. Differences were considered to be significant at p < 0.05. The statistical package SPSS version 19.0 was used for data analysis.

## Results and Discussion

As stated before, the purpose of this study was to develop and evaluate a vagino-adhesive propranolol hydrochloride gel with potential use in short-term contraception. To achieve this, various mucoadhesive polymers were utilized to prepare the gel formulations. Different tests (mentioned in the previous section) were performed on these formulations in order to gain a formulation with desirable characteristics. The results obtained would be presented and discussed in the following sections.

*Formulation and characterization of propranolol HCl gels*

In the process of formulating the vagino-adhesive propranolol HCl gels, it was observed that formulations F_19_ – F_28_ containing different concentrations of Na CMC and carbomers (C934 and C940) were not able to form gel in the aqueous solution containing propranolol hydrochloride. This observation could be attributed to the fact that carbomers; based on their anionic nature and large number of acidic groups available in their polymeric structure; tend to interact with cationic substances. Propranolol hydrochloride, which is a cationic drug, and carbomers interact ionically, giving rise to the formation of insoluble complexes which significantly affect drug release properties and mucoadhesiveness (23). The same phenomenon would stand for explanation of the interaction observed between Na CMC and the drug, as this polymer also possesses an anionic nature. Hence, formulations F_19_ – F_28_ were left out of further studies. Physical appearances and apparent viscosities of propranolol HCl gel formulations F_1_− F_18_ are given in Table 3. Formulations F_1_, F_5_, F_6_, F_10_, F_11_ and F_15 _had low and or relatively low apparent viscosities. A low-viscosity vaginal gel formulation is not as efficacious as a high-viscosity vaginal gel for use as a contraceptive gel, since rheological properties of spermicidal vaginal gels have considerable influence on their contraceptive success. As the consistency of the applied product increases, its efficacy may also increase as result of becoming more tenacious and more resistant to sperm migration, and consequently decreasing the capability of sperm to reach the site of fertilization (8).

Formulations F_4_, F_9_, F_14_ and F_18_ showed very high apparent viscosities and gels with lumps rather than smooth gels, were formed. Hence, all the aforementioned formulations were excluded from further studies. Among the rest of the formulated gels, formulations F_2_, F_3_, F_7_, F_8_, F_12_, F_13_, F_16_ and F_17_ which exhibited suitable apparent viscosities (relatively high or high) as well as good physical appearances, were used for further studies.

**Figure 3:**
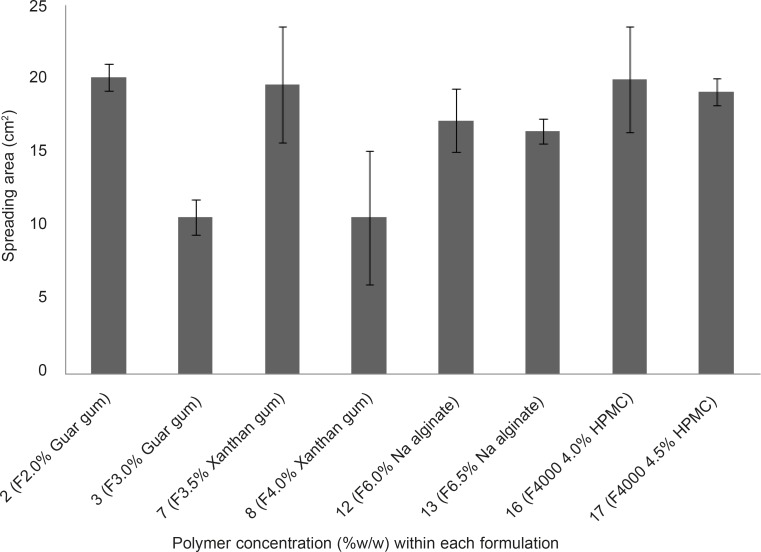
Comparison of spreading area of vagino-adhesive propranolol HCl gel formulations (F_2_, F_3_, F_7_, F_8_, F_12_, F_13_, F_16_ and F_17_). Data are expressed as mean ± SD (n = 3).

*Spreadability measurement of propranolol HCl gel formulations*

The spreading area of propranolol HCl gel formulations F_2_, F_3_, F_2_, F_8_, F_12_, F_13_, F_16_ and F_17_ was measured according to the technique explained earlier. The results are shown in Figure 3. The spreading areas of these formulations were in the following order: F_2 _> F_16 _> F_7_ > F_17_ > F_12_ > F_13_ > F_8_ > F_3_. Data obtained were statistically analyzed using one-way ANOVA, followed by Tukey post-hoc test. It was found that the difference in spreading area between the propranolol HCl gel formulation, showing the highest spreadability (F_2_, containing 2% w/w guar gum) and formulations F_3_ and F_8 _was statistically significant (p < 0.05), whereas this difference between the gel formulation F_2_ and all other tested formulations was statistically insignificant ( p > 0.05). Bachhave *et al.* developed a vaginal gel of fluconazole. They considered a spreading area of 40.69 cm^2^ as good spreadability (17), while the highest spreading area obtained in our study was 20.15 cm^2^ ± 0.92 which was found to be poor in comparison to that obtained for fluconazole vaginal gel. The reason for poor spreadability of all the developed propranolol HCl gel formulations was their high viscosities due to their high polymer concentration. However, in case of contraceptive vaginal gels, having high viscosity not only speaks in favor of better contraceptive efficacy, but also increases the mucoadhesivity and retention of the gel formulation in the vaginal canal (6, 8). 

**Table 3:** Physical appearance and apparent viscosities of propranolol HCl gel formulaions (F1−F18)*.*

**Formulation code**	**Physical appearance**	** Apparent viscosity**	**Formulation** **code**	**Physical appearance**	**Apparent viscosity**
F_1 _	Transparent, light green color, without lumps	Low	F_10_	Translucent, light yellow color, without lumps	Low
F_2_	Transparent, light green color, without lumps	Relatively high	F_11_	Translucent, light yellow color, without lumps	Relatively low
F_3_	Transparent, light green color, without lumps	high	F_12_	Translucent, light yellow color, without lumps	Relatively high
F_4_	Transparent, light green color, with lumps	Very high	F_13_	Translucent, light yellow color, without lumps	high
F_5_	Translucent, white color, without lumps	Low	F_14_	Translucent, light yellow color, with lumps	Very high
F_6_	Translucent, white color, without lumps	Relatively low	F_15_	Transparent, colorless, without lumps	Relatively low
F_7_	Translucent, white color, without lumps	Relatively high	F_16_	Transparent, colorless, without lumps	Relatively high
F_8_	Translucent, white color, without lumps	high	F_17_	Transparent, colorless, without lumps	high
F_9_	Translucent, white color, with lumps	Very high	F_18_	Transparent, colorless, with lumps	Very high

*Assessment of mucoadhesive strengths of propranolol HCl gels*

**Figure 4:**
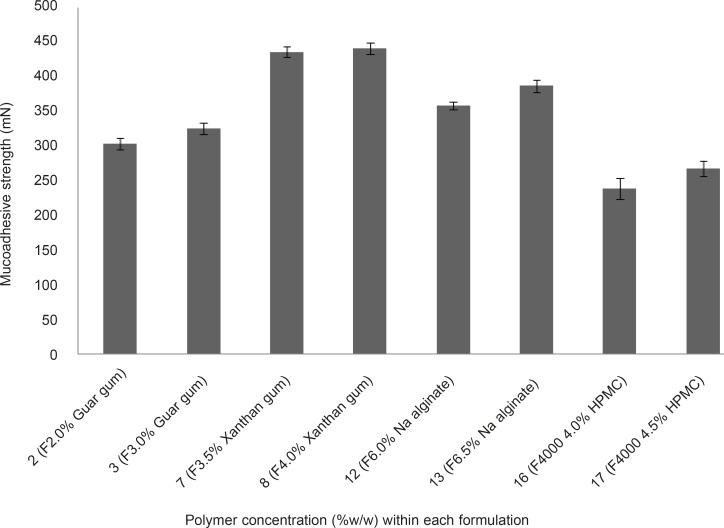
Mucoadhesive strength of vagino-adhesive propranolol HCl gel formulations. Data are expressed as mean ± SD

The mucoadhesive strengths of propranolol HCl gel formulations (F_2_, F_3_, F_7_, F_8_, F_12_, F_13_, F_16_ and F_17_) were examined and results are shown in Figure 4. It is evident from Figure 4 that, in case of formulations containing the same polymer but at different concentrations (*e.g.* F_2_ and F_3_, as compared together), when the concentration of the polymer increased the mucoadhesive strength escalated, accordingly. This phenomenon can be attributed to the fact that, at lower concentration of the polymer chains, there is an inadequate and unstable interaction between the polymer and the mucosal layer resulting in lower mucoadhesive properties (7). The mucoadhesive strengths observed, were in the following order: F_8 _> F_7 _> F_13_ > F_12_ > F_3_ > F_2_ > F_17_ > F_16_. Propranolol HCl gel formulations F_7_, F_8_ (containing 3.5 and 4.0% w/w xanthan gum, respectively), F_12_ and F_13_ (containing 6.0 and 6.5% w/w sodium alginate, respectively), showed statistically significant (p < 0.05, one-way ANOVA and Tukey post-hoc test) higher mucoadhesive strengths as compared to formulations F_12_, F_13_ (containing 2.0 and 3.0% w/w guar gum, respectively), F_16_ and F_17_ (containing 4.0 and 4.5% w/w HPMC 4000, respectively).This finding can be explained by the fact that the presence of charged functional groups in the polymer chains, which can be observed in xanthan gum and sodium alginate (presence of negatively charged carboxyl groups), may render them as polyelectrolytes and this has a marked effect on the strength of mucoadhesion due to the formation of strong hydrogen bonds between the polymer functional groups and the mucosal layer as compared to neutral polymers such as guar gum and HPMC. In general, anionic polyelectrolytes have been found to form stronger mucoadhesive bond when compared to the neutral polymers (7). Propranolol HCl gel formulations F_8_, F_7_, F_13_ and F_12_ showed the highest mucoadhesive strengths among all the tested formulations. However, formulations F_3_, F_8_, F_13_ and F_17 _were considered as the chosen formulations for further assessments. The reason for selecting these formulations, was to investigate how an individual type of polymer can affect the propranolol HCl release from the gel matrix.

*Determination of in-vitro drug release profiles from the chosen formulations*

**Figure 5:**
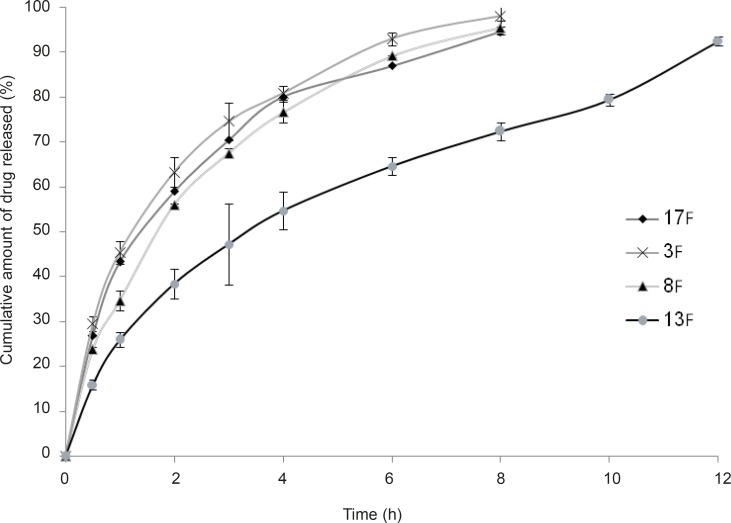
*In-vitro *release profiles of vagino-adhesive propranolol HCl gel formulations F3 ( Guar gum 3.0% w/w), F8 ( Xanthan gum 4.0% w/w), F13 ( Na alginate 6.5% w/w) and F17 ( HPMC 4000 4.5% w/w) at 37°C, citrate-phosphate buffer pH 4.5. Data are expressed as mean ± SD.

**Figure 6:**
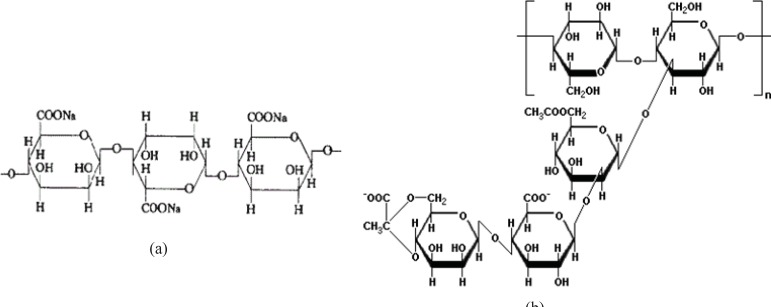
Chemical structures of sodiom alginate (a) and xanthan gum (b).

As stated in the previous section, propranolol HCl gel formulations F_3_, F_8_, F_13_ and F_17_ were chosen and their *in-vitro* drug release profiles were determined. The results obtained have been shown in Figure 5. As can be clearly observed in this figure, the drug release profile was slower from formulations containing anionic polymers such as formulations F_13_ and F_8_, which were contained of sodium alginate 6.5% w/w and xanthan gum 4% w/w, respectively. However, propranolol HCl gel formulation F_13_ released its drug content in a more retarded manner as compared to formulation F_8­_. This observation may be attributable to the formation of ionic complexes (without affecting the gel structure) between propranolol HCl (a cationic drug) and sodium alginate (an anionic polymer). The chemical structures of sodium alginate and xanthan gum are shown in Figure 6. As it can be clearly seen in this figure, sodium alginate possesses more anionic carboxylate groups in its structure (there’s one carboxyl group on each of the monomers), while xanthan gum has only two carboxyl groups in the terminal side of its polymeric chain. Moreover, sodium alginate had been used in a higher concentration in formulation F_13_ (6.5% w/w) as compared to xanthan gum in formulation F_8 _(4.0% w/w). Hence, formation of greater drug-polymer complexes and subsequently a slower drug release pattern would be deducible from the F_13_ formulation. Formulations F_3_, F_8_ and F_17_ released nearly 100% of their drug contents over a period of 8 h, while propranolol HCl gel formulation F_13_ released more than 85% of its drug content throughout a 12 h period. As could be seen in release profile of this formulation (F_13_), a burst release occurred in the first 2 h after the drug release was initiated. In this case, burst release can be considered as a desirable phenomenon, since an initial burst is assumed to provide immediate contraceptive effects, through causing the propranolol HCl concentration exceeding its IC50 value (0.3 mM) followed by a prolonged release which maintains drug concentration above the IC50 value with the potential of promoting contraceptive efficacy for an extended period of time.

*In-vitro drug release kinetic studies of the chosen formulations*

Data obtained from *in-vitro* drug release of the chosen propranolol HCl gel formulations F_3_, F_8_, F_13_ and F_17_ were fitted to various mathematical models including: zero order, first order, Higuchi and Korsemeyer-Peppas models. The drug release kinetic data for the above formulations are given in Table 4. The model that best fitted the release data of each propranolol HCl gel formulation was evaluated by the highest regression coefficient (R^2^). 

The zero order kinetic model (Equation 2) describes systems where the drug release rate is independent of its concentration. In contrast, first order kinetic model (Equation 3) is indicative of systems where drug release is a concentration dependent process. Higuchi’s model (Equation 5) describes the release of drugs from an insoluble matrix as the square root of a time dependent process, based on Fickian diffusion. Korsemeyer-Peppas model (Equation 5) is applicable when diffusion is the main drug release mechanism, while relating the drug release exponentially to the elapsed time. Data obtained from release profile of formulation F_3_, fitted best into Korsemeyer-Peppas model with an R^2 ^value of 0.9946, while the drug release profiles of formulations F_8_ and F_17_ could be explained by the first order kinetic model as their drug release data fitted best into this mathematical model (with R^2 ^values of 0.9972 and 0.9895, respectively). In case of formulation F_13_, the best fit with the highest regression coefficient (R^2 ^= 0.9955) was found to be with the Higuchi’s model.

By incorporating the first 60% of drug release data, mechanism of drug release can be peredicted using the Korsemeyer-Peppas model, where *n* is the release exponent, indicative of mechanism of drug release. The values of release exponent (*n*) for propranolol HCl gel formulations F_3_, F_8_, F_13_ and F_17 _were found to be 0.597, 0.614, 0.562 and 0.566, respectively. Hence, the mechanism of propranolol HCl release from all these formulations, would be based on anomalous (non-Fickian) diffusion (as described in Table 2) which appears to indicate a coupling of diffusion and erosion mechanisms, so-called anomalous diffusion and may indicate that the drug release is controlled by more than one process (20). In a previous study describing the release mechanism of domperidone from alginate-based beads, similar results were observed with *n* values ranging from 0.485 to 0.771. They also considered the corresponding *n *values to be indicative of anomalous release mechanism (24).

Among the chosen formulations tested for determination of drug release profile, only propranolol HCl gel formulation F_13 _could release its drug content throughout a 12 h period and the release pattern of this formulation was the most desirable as compared to other formulations. It also revealed a good mucoadhesive strength (386.97 ± 9.31 mN). Hence, propranolol HCl gel formulation F_13_ was considered as the final formulation and underwent complementary studies including determination of drug content and assessment of the duration of mucoadhesion.

**Table 4:** Results of kinetic studies conducted on vagino-adhesive propranolol HCl gel formulations F3, F8, F13 and F17, based on various mathematical models fitted.

**Formulation code**	**Zero order**		**First order**		**Higuchi**		**Korsemeyer-Peppas**	
	***K***_0 _ ***(h*** ^-1^ ***)***	***R*** ^2^	***K***_1 _ ***(h*** ^-1^ ***)***	***R*** ^2^	***K***_H _ ***(h*** ^-1/2^ ***)***	***R*** ^2^	**Release exponent ** ***(n)***	***R*** ^2^
**F**_3_	10.724	0.7995	0.470	0.9871	35.536	0.9704	0.597	0.9967
**F**_8_	11.029	0.8564	0.380	0.9972	35.586	0.9856	0.614	0.9950
**F**_13_	6.700	0.9107	0.180	0.9478	26.180	0.9955	0.562	0.9927
**F**_17_	10.360	0.8044	0.340	0.9895	34.241	0.9712	0.566	0.9849

*Determination of drug content within the final gel formulation*

As there is no pharmacopeial monograph for vagino-adhesive proranolol HCl gel, the range of 90-110% of the claimed label which is applicable for most pharmaceutical dosage forms, was considered for drug content evaluation of the final gel formulation. The propranolol HCl content of this formulation (F_13_) was found to be 101.05% ± 0.106 (n = 3, mean ± SD) of the theoretical value (1.6% w/w), which complies with pharmacopeial specifications for drug content. Hence, it can be concluded that a proper method had been used for drug content analysis and the drug had been evenly distributed within the gel matrix.

*Determination of the duration of mucoadhesion of the final formulation*

As it had been stated in a previous study (19), in most cases only the mucoadhesive strength of a newly formulated mucoadhesive system is evaluated, despite the fact that it is possible that a mucoadhesive system could initially adhere strongly to the mucosal surface but in long term it could quickly overhydrate and get displaced from the site of adhesion. Hence, investigating both these parameters is critical in the development of an effective mucoadhesive dosage form. In order to measure the duration of mucoadhesion of the final formulation (F_13_), a 7.0 g weight was applied to the upper platforms of the apparatus shown in Figure 2. The results indicated that, by applying a maximum weight of 7.0 g, the gel could remain attached to the vaginal mucosa for more than 10 h. In this study the gel was fully immersed in an aqueous environment in order to prevent it from dryness and resembles the presence of vaginal secretions. However, this condition could result in a quicker hydration and faster disruption of the gel, in contrast to vaginal canal which contains less fluid. At the end, it should be noted that in both the evaluation of mucoadhesive strength and duration of mucoadhesion tests, separation of the gel from the mucosal surface appeared to be a result of a cohesive failure within the gel structure. Therefore, the mucosal surface was found to be coated with a rather thin layer of gel at the end of each test (*i.e.* when gel mucosa separation took place). This finding would mean that in the *in-vivo* condition, a thin layer of gel could still remain in contact with the vaginal mucosa, even after the main bulk of the gel applied has been washed away by vaginal secretions, prolonging the residence time of the gel and allowing further drug release from it.

## Conclusions

The most widely used mucoadhesive vaginal drug delivery systems are gels. Gels are easy to manufacture, comfortable and have the ability to spread onto the surface of vaginal mucosa. The employment of mucoadhesive polymers can improve time contact with the mucosal surface, delaying the loss of formulation and hence, prolonging the effect. 

In this study various mucoadhesive polymers were evaluated for the preparation of a contraceptive, vagino-adhesive propranolol HCl gel. Results of the present research demonstrated that, the gel formulation F_13_ (containing sodium alginate 6.5% w/w and 1.6% w/w propranolol HCl, beside the necessary exipients in an aqueous vehicle) was considered as the most desirable formulation, since it exhibited appropriate mucoadhesive properties while having the potential of providing an immediate contraceptive effect, followed by a prolonged drug release which is assumed to render longer contraceptive efficacy. Further investigations also confirmed that this gel formulation is capable of adhering to the model mucosa for more than 10 h. Propranolol hydrochloride, due to being devoid of mucosal toxicity observed with frequent use of N-9, has been proposed as an ideal alternative to this spermicide. However, further *in-vivo* studies are required to evaluate the efficacy of contraceptive vagino-adhesive propranolol HCl gel.
